# Bidirectional associations among positive affect, anhedonia and meaning in life during major depressive episode: ecological momentary assessment study in unipolar and bipolar individuals and healthy controls

**DOI:** 10.1192/bjo.2025.10067

**Published:** 2025-07-07

**Authors:** Heidi Ka Ying Lo, Roger S. McIntyre, Iris Wai Tung Tsui, Fiona Yan Yee Ho, Ting Kin Ng, Corine Sau Man Wong, Suet Ying Yuen, Chit Tat Lee, Chun Yin Poon, Inez Myin-Germeys, Ka Fai Chung

**Affiliations:** Department of Psychiatry, School of Clinical Medicine, LKS Faculty of Medicine, The University of Hong Kong, Hong Kong; Department of Psychiatry, University of Toronto, Canada; Department of Pharmacology, University of Toronto, Canada; Department of Psychology, The Chinese University of Hong Kong, Hong Kong; Department of Psychology, Lingnan University, Hong Kong; School of Public Health, LKS Faculty of Medicine, The University of Hong Kong, Hong Kong; Department of Psychiatry, Queen Mary Hospital, Hong Kong; Center of Contextual Psychiatry, Department of Neurosciences, KU Leuven, Belgium

**Keywords:** Experience sampling method (ESM), ecological momentary assessment (EMA), depression, bipolar disorder, anhedonia

## Abstract

**Background:**

Diagnostic accuracy is an unmet need for major depressive disorder (MDD) and major depressive episode (MDE) in bipolar disorder. Very limited research has evaluated bipolar disorder/MDE and MDD using ecological momentary assessment (EMA) time-series data.

**Aims:**

We aimed to examine differentiating phenomenological characteristics in positive affect dynamics, and temporal relationships with pleasure towards current activity and meaning in life (MIL), among MDD, MDE/bipolar disorder and healthy controls using EMA.

**Method:**

Participants (*N* = 88, mean age 28.7 years, 69% female), including individuals with MDD (*n* = 29) and MDE/bipolar disorder (*n* = 29) and healthy controls (*n* = 30), were assessed for positive affect, pleasure and MIL 5 times daily over a 2-week period. Multilevel modelling analysis was conducted, with estimation of first-order autoregressive model structure and time-lagged relationship between pleasure and positive affect.

**Results:**

From 4632 EMA observations, positive affect dynamics (inertia, variability and instability) did not differ significantly across groups (all *P* > 0.05). Although all groups demonstrated a bidirectional relationship between positive affect and pleasure, for MDE/bipolar disorder, both pleasure_*t* − 1_ (*β* = −0.11, *t*[51.09] = −2.31, *P* = 0.025) and positive affect_*t* − 1_ (*β* = −0.13, *t*[56.54] = −2.30, *P* = 0.025) predicted subsequent MIL less significantly than for MDD and healthy controls.

**Conclusion:**

Individuals with MDE/bipolar disorder, but not MDD, had less self-reported MIL from positive affect and pleasure. There is little evidence that emotional experience alone characterises the pathophysiology between MDD and MDE/bipolar disorder; such investigation may be limited by within-group heterogeneity. Our findings provide a new perspective on using a time-series approach beyond bimodal measures in EMA to differentiate bipolar disorder/MDE and MDD.

Major depressive disorder (MDD) and bipolar disorder both present with major depressive episodes (MDEs) involving considerable symptomatic overlap, which poses conceptual challenges in terms of their diagnostic boundaries and practical difficulties in optimisation of treatment.^1^ The lack of detailed understanding regarding the distinct psychopathological characteristics of MDD and MDE/bipolar disorder hampers early identification and delays the implementation of guideline-recommended treatment.^1,2^ MDE/bipolar disorder is highly heterogenous between and within individuals and its pleomorphic clinical features further challenge differentiation, especially when considering the real-world ecological validity of the psychopathology of MDE/bipolar disorder versus MDD.^3^ Cognitive-emotional evaluation, the process by which individuals assess and interpret their emotional experiences, is disrupted in both MDD and bipolar disorder.^4^ These disturbances are central to understanding the pathophysiology of mood disorders^5^ and are crucial for developing effective treatment strategies. The construct of meaning in life (MIL) has typically been considered a cognitive aspect of well-being, encompassing goal-driven engagement and the subsequent sense of satisfaction brought forth. Research consistently illustrates that MIL is strongly associated with positive affect:^6^ experimentally induced positive mood increased MIL measures, with positive affect being a predictor of MIL.^6^ This relationship is particularly relevant in the context of mood disorders such as MDD and bipolar disorder, where disruptions in the cognitive-emotional loop are prevalent.^5^ Anhedonia, a core symptom of MDE that involves a diminished ability to experience pleasure, particularly impairs the reward processing essential for goal-driven activities, thereby undermining the perception of MIL.^7,8^ Further building on these insights, our study explores how MIL, beyond its association with positive affect, also acts as a protective factor and potential tailoring strategy against the debilitating effects of mood disorders.^9^

## Ecological momentary assessment of positive affect in MDD and bipolar disorder during MDE

Ecological momentary assessment (EMA) has emerged as a promising approach in translating such processes in day-to-day cognitive-emotional symptom evaluation, which enables researchers to capture real-time, serial, intra-individual symptom data within natural environments, providing them with a dynamic tool to understand mood disorder intricacies more effectively.^3,10^ EMA is demonstrated as a feasible and valid symptom assessment approach with good adherence and acceptability, and has been increasingly applied in cognitive-emotional symptom research – in particular, for emotional experiences and cognitive appraisals.^10,11^ For MDD and bipolar disorder, the findings on affect dynamics are mixed. Given the pivotal role of positive affect in mental health, it is crucial to investigate its dynamics, particularly in the context of mood disorders.^12^ While some EMA studies^13,14^ found no difference in affect dynamics between MDD and healthy controls, others (e.g. refs^15–17^) have indicated that MDD had greater variability and instability in regard to positive affect. Some studies on affect dynamics in bipolar disorder reported higher variability,^18,19^ while others suggested no difference in affect dynamics as compared with healthy controls.^20,21^ A notable limitation of these studies is the lack of specification of the clinical phase, which is critical because hypomanic or mixed states in bipolar disorder may inherently induce greater affective variability and instability. In addition, previous EMA studies seldom directly compared MDD and bipolar disorder, and none were conducted in non-Western countries. There is only one published study^22^ assessing current MDE in bipolar disorder and MDD, which found no differences in their affect dynamics. In addition, further idiographic insight into prediction with the dynamic patterns of depression necessitates the methodology of combining time-series data with EMA data through a temporal relationship, which uses Granger causality to delineate lagged relationships between dynamic experiences from one time point (*t* – 1) to the next (*t*).^23^ To the best of our knowledge, no work has yet investigated the time-lagged relationship between emotional and cognitive experiences in MDD and bipolar disorder during MDE.

## Study aims and hypotheses: investigating EMA positive affect, anhedonia and MIL in MDD and bipolar disorder

In the current study, we aimed to systematically examine momentary manifestations of cognitive-affective symptoms in Chinese subjects with bipolar disorder and MDD in their everyday life in Hong Kong, utilising an EMA that evaluats three well-defined constructs encompassing (a) affective experiences (operationalised as positive affect level, dynamics of positive affect and negative affect); (b) anhedonia (operationalised as pleasure/motivation towards current event/activity); and (c) cognitive appraisal (MIL). Because it was demonstrated that positive affect, specifically, more than its counterpart negative affect, predicts reward and emotional processing in depression,^24^ our analysis focuses primarily on positive affect. Based on previous literature, including EMA research, we hypothesised that, compared with healthy controls, both MDD and bipolar disorder subjects in their MDE states would have (a) lower positive affect levels; (b) more unstable positive affect dynamics, specifically in terms of inertia, variability and instability; (c) positive affect that less prospectively predicts the level of pleasure/motivation towards current event/activity at the next sampling occasion, and vice versa; and (d) an impaired bidirectional relationship between emotional experiences (positive affect/pleasure) and appraisal (MIL) (i.e. increases in positive affect/pleasure were less effectively predictive of subsequent MIL, albeit people find it harder to connect with life’s activities that were previously satisfying/rewarding).

## Method

### Participants

Ninety-nine participants were recruited for the study from August 2023 to January 2024. The sample consisted of 29 participants with MDD, 29 with MDE/bipolar disorder and 30 healthy controls. The three groups were matched for age (±3 years), gender and education. Participants with MDD and MDE/bipolar disorder were out-patients at a regional psychiatric clinic in Hong Kong; healthy controls were recruited through mass emails and word of mouth. All participants had been on a stable medication regime for >4 weeks before the study. Eleven participants were excluded from the analysis: three due to scheduling conflicts resulting in missing baseline assessment, four due to technical difficulties in downloading/using the m-Path application (see below) and four due to one third EMA compliance or below, as adopted by other studies.^25^ There were no significant differences in age, gender and total years of education between participants who were included and excluded from the analysis. The final sample consisted of 88 participants. The study was reviewed and approved by the Institutional Review Board of The University of Hong Kong/Hospital Authority Hong Kong West Cluster (no. HKWC-2023-207). Written informed consent was obtained from all participants. Informed consent for participants aged <18 years was accompanied by their parent/legal guardian, from whom written informed consent was also collected.

For the MDD and MDE/bipolar disorder groups, the inclusion criteria were as follows: (a) Hong Kong Chinese aged between 14 and 65 years; (b) presence of current MDD or bipolar disorder based on the Chinese bilingual Structured Clinical Interview for Diagnostic and Statistical Manual of Mental Disorders for DSM-IV (SCID-I); (c) a score of ≥7 on the Hamilton Depression Rating Scale (HDRS), indicating at least mild depression; and (d) a score of ≤12 on the Young Mania Rating Scale (YMRS), indicating the absence of hypomanic or mixed episode for the bipolar disorder group. For the healthy controls group, the inclusion criteria were as follows: (a) Hong Kong Chinese aged between 14 and 65 years; (b) HDRS total score <7, indicating minimal or no depressive symptoms; and (c) absence of current or lifetime significant psychiatric disorders based on SCID-I. The exclusion criteria for all groups included the inability to read Chinese, use smartphones or provide informed consent; intellectual disability; personality disorders; neurological or neurodevelopmental disorders; history of head injuries; and having a score of ≥4 on any 1 of the 3 items on the Positive and Negative Syndrome Scale (PANSS: P1 delusion, P2 conceptual disorganisation, P3 hallucination). Diagnoses of MDD and bipolar disorder were based on medical records and then ascertained using the SCID-I interview.

### Measures

#### Baseline assessment

Baseline assessments comprised four sections, including sociodemographic characteristics, clinical symptom profiles, psychosocial measures – including personality/resilience/loneliness – and functioning. In the current analysis we focused on basic sociodemographics including age, gender, educational level, employment, marital status and core symptom measures: depressive, manic and anhedonia symptoms. Depressive and manic symptoms were measured using HDRS and YMRS, respectively. Anhedonic symptoms were assessed using the Chinese version of the Snaith–Hamilton Pleasure Scale (C-SHAPS), a 14-item, self-rated scale. C-SHAPS demonstrated good psychometric properties in both individuals with depression and healthy controls.

#### EMA following baseline assessment

All eligible participants completed a baseline assessment and a 14-day EMA. Participants received a training session and a standardised instructional manual about the 14-day EMA data collection. EMA included 15 questions concerning levels of affect, activity, pleasure/motivation towards current activity (operationalised as anhedonia) and MIL (Table 1). EMA was delivered via the m-Path application, an experience-sampling platform developed by Katholieke Universiteit Leuven.^26^ The m-Path platform has been used to investigate various constructs including smartphone use, momentary emotions and work-related stress, demonstrating high usability and acceptability among practitioners and users alike.^11,26^ EMA was conducted over 14 consecutive days, emitting 5 signals daily. At each signal, participants received a notification that directed them to an identical set of questions written in Chinese. Participants who had not responded to EMA would receive two reminders, 15 and 30 min after the signal had elapsed, and the questionnaire would expire after 1 h. Responses were time-stamped by the m-Path application. All but activity measurements were rated on a sliding scale (0 being lowest and 100 highest). The monetary incentive was scaled to the level of compliance, with a maximum of USD36. Participants received daily reminder messages and technical support from research staff during the 14-day EMA period.

**Table 1:** Ecological momentary assessment questions

EMA variables	Description
PA	The mean of four PA variables: ‘How happy are you now?’, ‘How content are you now?’, ‘How relaxed are you now?’ and ‘How energetic are you now?’, rated using a sliding scale from 0 (not at all) to 100 (extremely). Items were selected with reference to the positive and negative affect schedule in accordance with other EMA studies.^22^
NA	The mean of four NA variables: ‘How nervous are you now?’, ‘How tired are you now?’, ‘How sad are you now?’ and ‘How angry are you now?’, rated on a sliding scale from 0 (not at all) to 100 (extremely). Items were selected with reference to the positive and negative affect schedule in accordance with other EMA studies.^22^ Four PA items (happy, energetic, relaxed and content) and four NA items (sad, tired, nervous and irritated) were included and ordered alternately (i.e. one PA item followed by one NA item).
PA variability	Standard deviation of momentary PA per participant.
PA instability	The mean squared successive difference of momentary PA between beeps per participant.
PA inertia	Autocorrelation of momentary PA per participant. An autocorrelation score was computed between two consecutive beeps, then an overall autocorrelation score was computed by averaging all scores per participant.
Meaning in life	Score for the question, ‘At this moment, how much would you say your life is meaningful?’, which was written with reference to the Meaning in Life Questionnaire and rated using a sliding scale from 0 (not at all) to 100 (extremely).
Current activity	Answer to the multiple-choice question, ‘What are you doing now?’ Answers differ by goal directed (e.g. sports, chores) and non-goal directed (e.g. nothing in particular), referenced from previous EMA studies.
Current company	Answer to the multiple-choice question, ‘Who are you with now?’ Answers differ by the type of company (e.g. family members, strangers, alone).
Anhedonia (operationalised as pleasure/motivation towards current activity)	The mean of four anhedonia variables: ‘Just before receiving this message, how much did you anticipate doing your current activity?’, ‘How much are you enjoying your current activity?’, ‘How much effort are you exerting on your current activity?’ and ‘How motivated are you to seek for or to do your current activity?’, rated using a sliding scale from 0 (not at all) to 100 (extremely). Items were selected according to recent conceptualisations of anhedonia as a dimensional construct with both hedonic and motivational components, with reference to a previous EMA study.^4^ Four questions tapped into participants’ anticipatory and consummatory anhedonia, as well as current motivation and effort to attain rewards.

EMA, ecological momentary assessment; PA, positive affect; NA, negative affect.

### Statistical analysis

In our statistical analysis utilised to test the proposed hypotheses, we employed a multilevel analysis approach. In our multilevel analysis, we addressed missing data using restricted maximum likelihood (REML) estimation, as implemented in the lmer() function of the R package lme4. This approach leverages all available data points and is appropriate under the assumption that data are missing at random (MAR). For hypothesis 1, we utilised multilevel analysis to assess group differences in positive affect level, with each momentary observation (level 1) nested within participants (level 2). This involved computing mean positive and negative affect levels by averaging responses to four items at each time point.^27^ In addressing hypothesis 2, each positive affect dimension was first calculated for every participant before entering the values into a linear regression model, with group as the independent variable. MDD and MDE/bipolar disorder were dummy-coded categorical variables used to assess group differences with reference to the healthy controls group. Positive affect inertia was operationalised by computing the autocorrelation of participants’ positive affect level to obtain a single autocorrelation value. Positive affect variability and stability were measured using standard deviation and mean square successive difference (MSSD), respectively. Hypotheses 3 and 4 proposed a bidirectional relationship between positive affect, anhedonia and MIL. We anticipated differences between the MDD and MDE/bipolar disorder groups and healthy controls, and adopted a multilevel modelling analysis with a first-order autoregressive model structure.^23^ This approach allowed us to examine the time-lagged relationship of the predictive value of one variable at a preceding time point, *t* − 1, on another at the subsequent time point, *t*. The presence of group differences in time-lagged relationships was implicated by the statistical significance of the group × preceding variable_*t* − 1_ interaction term. All analyses were conducted using R (v. 4.0.3 for macOS, R Foundation for Statistical Computing, Vienna, Austria; see https://cran.r-project.org/bin/macosx/), specifically the lm() and lmer() packages. A *P*-value cut-off of 0.05 was employed for all analyses.

## Results

### Sample characteristics

EMA compliance rate (defined as the percentage of prompts to which participants responded) for the study was 75.2% (s.d. = 25.06). As shown in Table 2, 88 participants (27 male, 31%) were included in the final EMA analysis, comprising 29 in the MDD group (mean age 27.5 years, s.d. = 10.1), 29 in the MDE/bipolar disorder group (mean age 30.4 years, s.d. = 10.5) and 30 in the healthy controls group (mean age 28.3 years, s.d. = 9.7). There was no significant difference in sociodemographic characteristics across groups. In terms of clinical characteristics, the MDD and MDE/bipolar disorder groups exhibited significantly higher levels of depression severity than the healthy controls group (s.d. = 4.35, *P* < 0.001), with HDRS scores (MDD, 13.4, s.d. = 5.6; MDE/bipolar disorder, 13.6, s.d. = 4.8). Similarly, the MDD and MDE/bipolar disorder groups had significantly higher levels of anhedonia as compared with the healthy controls group, with C-SHAPS scores for MDD of 14.0 (s.d. = 9.5) and, for MDE/bipolar disorder, 12.8 (s.d. = 6.2).

**Table 2:** Sample characteristics of participants, presented as *n* (%) or *M* (s.d.) by group

	MDD (*n* = 29)		MDE/BD (*n* = 29)^a^		HC (*n* = 30)		Group comparison
Characteristics	Mean (*n*)	s.d. (%)	Mean (*n*)	s.d. (%)	Mean (*n*)	s.d. (%)	
Age (years)	27.5	10.1	30.4	10.5	28.3	9.7	*F*(2, 93) = 0.44, *P* = 0.64
Gender (male)	6	20.7	9	31.0	12	40	*χ* ^2^(2) = 2.59, *P* = 0.27
Sociodemographic characteristics							
Highest education level							*χ* ^2^(14) = 19.68, *P* = 0.14
Secondary school	7	24.1	2	6.9	4	13.3	
Vocational training/higher diploma	7	24.1	7	24.1	0	0	
Bachelor level	13	44.8	18	62.1	18	60	
Postgraduate level	2	6.9	2	6.9	8	26.7	
Employment status							*χ* ^2^(10) = 16.22, *P* = 0.09
Full-time working/studying	21	72.4	22	75.9	29	96.7	
Part-time working/studying	5	17.2	4	13.8	0	0	
Unemployed/not studying	3	10.3	2	6.90	1	3.3	
Marital status							*χ* ^2^(4) = 4.38, *P* = 0.36
Unmarried	21	72.4	23	79.3	28	93.3	
Married	7	24.1	3	10.3	2	6.7	
Divorced/widowed	1	3.5	2	6.9	0	0	
Clinical characteristics							
HDRS score	13.4^b^	5.6	13.6^b^	4.8	2.9^c^	4.4	*F*(2,85) = 45.44, *P* < 0.001**
YMRS score	--	--	2.9	3.3	--	--	
C-SHAPS score	14.^b^	9.5	12.8^b^	6.2	8.4^c^	6.6	*F*(2,83) = 4.25, *P* = 0.02*

C-SHAPS, Chinese version of the Snaith–Hamilton Pleasure Scale; HC, healthy controls; HDRS, Hamilton Depression Rating Scale; YMRS, Young Mania Rating Sale; MDD, major depressive disorder; MDE/BD, major depressive episode with bipolar disorder.

a Information about employment and marital status of one participant in the bipolar group was unrecorded.

b,c Means in the same row that do not share a superscript differ at *P* < 0.05 in post hoc comparisons.

*P < 0.05, **P < 0.001.

### Momentary positive affect level, inertia, variability and instability

Multilevel modelling analysis revealed that the positive affect level was lower in the MDD group (*B* = −7.39, *t*[85.31] = −1.87, *P* = 0.07) and MDE/bipolar disorder group (*B* = −7.64, *t*[85.34] = −1.93, *P* = 0.06) compared with the healthy controls group, but this difference marginally did not reach the statistical significance level (Table 3). There was no significant difference in regard to negative affect level, or to positive affect inertia, variability and instability, across groups.

**Table 3:** Multilevel modelling analysis of PA/NA level and PA inertia, variability and instability

Predictors	*B*	s.e.	d.f.	*t*	*P*
PA					
(intercept)^a^	59.49	2.78	85.78	21.39	
MDD	−7.39	3.96	85.31	−1.87	0.066*
MDE/BD	−7.64	3.96	85.34	−1.93	0.057*
NA					
(intercept)^a^	29.29	2.55	85.09	11.48	
MDD	0.28	3.64	84.73	0.08	0.940
MDE/BD	−0.46	3.64	84.76	−0.13	0.900
PA inertia (autocorrelation)					
(intercept)^a^	0.30	0.04	2.85	7.88	
MDD	−0.03	0.05	–	−0.60	0.548
MDE/BD	−0.03	0.05	–	−0.62	0.535
PA variability (s.d.)					
(intercept)^a^	14.61	1.03	2.85	14.56	
MDD	1.07	1.43	–	0.75	0.456
MDE/BD	−2.06	1.43	–	−1.44	0.154
PA instability (MSSD)					
(Intercept)^a^	371.17	45.69	2.85	8.12	
MDD	50.77	65.17	–	0.08	0.438
MDE/BD	−102.38	65.17	–	−1.57	0.120

MDD, major depressive disorder; MDE/BD, major depressive episode with bipolar disorder; MSSD, mean squared successive differences; PA, positive affect; NA, negative affect.

The three groups are the healthy control group, MDD and MDE/BD; the healthy control group is a reference group.

a Group comparison was conducted in relation to the healthy control group.

*P < 0.10.

### Time-lagged relationships between momentary pleasure/motivation towards current activity and positive affect level

As shown in Table 4, pleasure_*t* − 1_ was related to subsequent positive affect level_*t*_ (*B* = 0.09, *t*[41.30] = 4.38, *P* < 0.001). Conversely, positive affect level_*t* − 1_ significantly predicted subsequent pleasure_*t*_ (*B* = 0.17, *t*[39.67] = 5.44, *P* < 0.001). However, the analysis indicated no significant differences across groups, suggesting that the impact of prior pleasure on subsequent positive affect, and vice versa, was consistent across the groups.

**Table 4:** Time -lagged relationships between momentary PA and pleasure/motivation towards current activity

Predictors	*B*	s.e.	d.f.	*t*	*P*
Outcome, PA_*t*_					
(intercept)	41.86	3.34	46.27	12.53	
PA_*t* − 1_	0.26	0.03	42.33	9.23	<0.001***
Pleasure_*t* − 1_	0.09	0.02	41.30	4.38	<0.001***
MDD	−12.42	3.87	39.51	−3.21	0.003**
MDE/BD	−9.94	4.05	38.20	−2.46	0.019*
Pleasure_*t* − 1_ × MDD	0.07	0.05	30.50	1.35	0.188
Pleasure_*t* − 1_ × MDE/BD	0.05	0.06	35.20	0.96	0.342
Outcome, pleasure_*t*_					
(intercept)	38.06	4.67	17.24	8.15	
Pleasure_*t* – 1_	0.22	0.03	51.13	7.66	<0.001***
PA_*t* – 1_	0.17	0.03	39.67	5.44	<0.001***
MDD	1.38	6.43	16.74	0.22	0.832
MDE/BD	−4.92	6.54	17.26	−0.75	0.462
PA_*t* − 1_ × MDD	−0.09	0.07	20.81	−1.23	0.232
PA_*t* − 1_ × MDE/BD	−0.03	0.07	22.70	−0.39	0.700

MDD, major depressive disorder; MDE/BD, major depressive episode with bipolar disorder; PA, positive affect.

*P < 0.05, **P < 0.01, ***P < 0.001.

### Time-lagged relationship between momentary pleasure/motivation towards current activity, positive affect level and MIL

As shown in Table 5, initial cross-sectional analysis revealed a strongly positive association between MIL and pleasure (*B* = 0.56, *t*[4041.52] = 26.36, *P* < 0.001), indicating that MIL was related to pleasure level. Similarly, MIL was positively associated with positive affect (*B* = 0.64, *t*[4041.79] = 30.27, *P* < 0.001), indicating that higher MIL was linked to increased positive affect. The interaction terms revealed significant differences across groups. Notably, the MDD and MDE/bipolar disorder groups exhibited less synchrony to MIL, in terms of both pleasure (MDD group, *B* = −0.07, *P* = 0.016; MDE/bipolar disorder group, *B = −*0.10, *P* = 0.002) and positive affect (MDD group, *B* = −0.08, *P* = 0.009; MDE/bipolar disorder group, *B* = −0.07, *P* = 0.021), compared with the healthy controls group. The findings indicate that MIL and positive affect and pleasure were significantly related; however, this relationship was less marked in the MDD and MDE/bipolar disorder groups than in the healthy controls group.

**Table 5:** Cross-sectional and time-lagged relationships between PA, MIL and pleasure/motivation towards current activity

Predictors	*B*	s.e.	d.f.	*t*	*P*
Relationship between MIL and pleasure					
(intercept)	31.15	2.91	130.43	10.67	
Pleasure	0.56	0.02	4041.52	26.36	<0.001****
MDD	−6.39	4.09	121.58	−1.56	0.121
BD	−0.51	4.15	124.27	−0.12	0.903
Pleasure × MDD	−0.07	0.03	4040.12	−2.40	0.016**
Pleasure × MDE/BD	−0.10	0.03	4060.08	−3.08	0.002***
Relationship between MIL and PA					
(intercept)	27.79	2.84	128.41	9.79	
PA	0.64	0.02	4041.79	30.27	<0.001****
MDD	−4.61	3.98	119.28	−1.12	0.249
BD	−1.78	4.05	124.02	−0.44	0.662
PA × MDD	−0.08	0.03	4043.01	−2.61	0.009***
PA × MDE/BD	−0.07	0.03	4060.28	−2.31	0.021**
Reactivity of pleasure_*t*_ towards MIL_*t* – 1_					
(intercept)	39.05	5.73	5.55	6.82	
Pleasure_*t* – 1_	0.22	0.02	108.26	12.08	<0.001****
MIL_*t* – 1_	0.18	0.04	15.00	4.62	<0.001****
MDD	−1.33	7.88	5.67	−0.17	0.872
BD	−11.61	8.10	5.70	−1.43	0.204
MIL_*t* – 1_ × MDD	−0.01	0.10	6.48	−0.15	0.889
MIL_*t* – 1_ × MDE/BD	0.07	0.10	6.59	0.66	0.531
Reactivity of PA_*t*_ towards MIL_*t* – 1_					
(intercept)	35.89	3.32	85.95	10.82	
PA_*t* – 1_	0.25	0.03	62.09	9.76	<0.001****
MIL_*t* – 1_	0.16	0.03	31.72	4.96	<0.001****
MDD	−5.43	4.32	67.32	−1.26	0.213
BD	−7.89	4.47	67.53	−1.76	0.082*
MIL_*t* – 1_ × MDD	0.01	0.06	71.74	0.24	0.811
MIL_*t* – 1_ × MDE/BD	0.04	0.06	76.03	0.67	0.502
Reactivity of MIL_*t*_ towards pleasure_*t* – 1_					
(intercept)	39.49	3.99	44.90	9.89	
MIL_*t* – 1_	0.27	0.03	50.14	8.56	<0.001****
Pleasure_*t* – 1_	0.10	0.02	45.35	4.68	<0.001****
MDD	−5.40	5.03	39.76	−1.07	0.289
BD	0.29	5.09	41.28	0.06	0.954
Pleasure_*t* – 1_ × MDD	−0.05	0.05	41.94	−0.99	0.327
Pleasure_*t* – 1_ × MDE/BD	−0.11	0.05	51.09	−2.31	0.025**
Reactivity of MIL_*t*_ towards PA_*t* – 1_					
(intercept)	38.84	4.28	43.54	9.08	
MIL_*t* – 1_	0.26	0.03	52.14	8.12	<0.001****
PA_*t* – 1_	0.11	0.03	65.64	4.16	<0.001****
MDD	−3.12	5.54	39.23	−0.56	0.577
BD	1.37	5.62	41.32	0.24	0.809
Pleasure_*t* – 1_ × MDD	−0.07	0.06	47.78	−1.30	0.199
Pleasure_*t* – 1_ × MDE/BD	−0.13	0.06	56.54	−2.30	0.025**

MDD, major depressive disorder; MDE/BD, major depressive episode with bipolar disorder; MIL, meaning in life; PA, positive affect.

*P < 0.10, **P < 0.05, ***P < 0.01, ****P < 0.001.

**Fig. 1:**
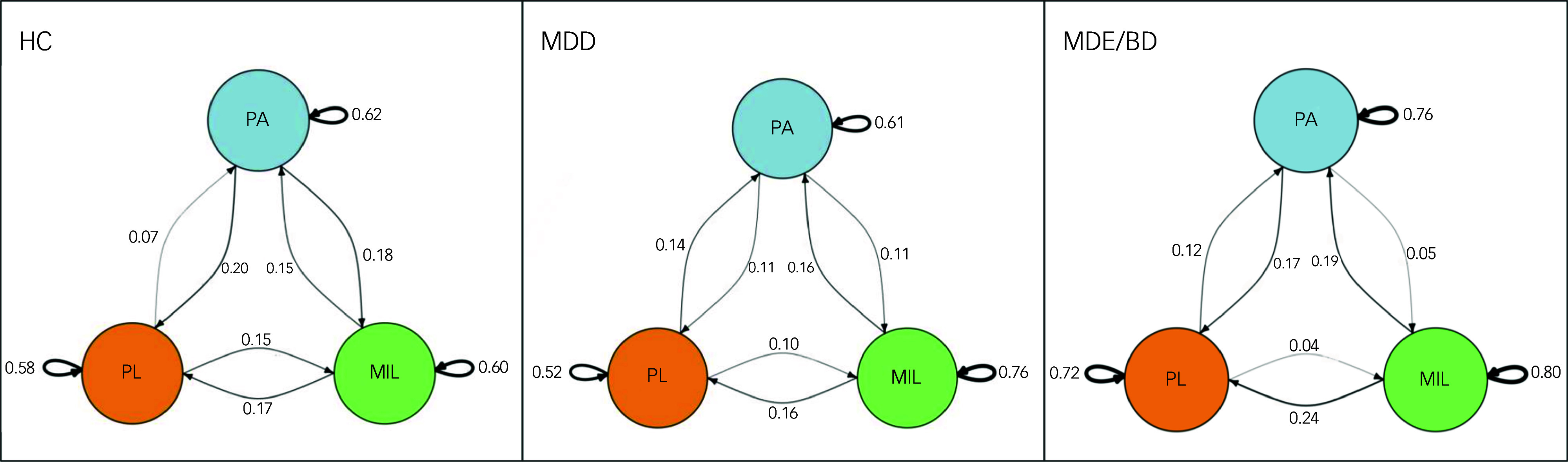
Graphic display of the cross-lagged model estimating the temporal association of PA, MIL and PL among participants in each group, HC, MDD and MDE/BD, using the igraph package. Curved arrows represent autocorrelations and straight arrows represent the strength of time-lagged relationships (e.g. PA_*t* – 1_ towards Pl_*t*_, PL_*t* – 1_ towards MIL_*t*,_), with the arrowed variable measured at the subsequent time point (i.e. time point_*t*_). Thicker edges represent stronger connections: the thinner the edge, the weaker the connection. HC, healthy controls; MDD, major depressive disorder; MDE/BD, major depressive episode with bipolar disorder; MIL, meaning in life; PA, positive affect; PL, pleasure.

Time-lagged analyses explored the bidirectional relationship between these constructs (Table 5). Figure 1 displays EMA visualisation of the cross-lagged model estimating the temporal associations of momentary positive affect, pleasure and MIL in each group – healthy controls, MDD and MDE/bipolar disorder. MIL_*t* − 1_ was significantly associated with pleasure_*t*_ (*B* = 0.18, *t*[15.00] = 4.62, *P* < 0.001) and positive affect_*t*_ (*B* = 0.16, *t* [31.72] = 4.96, *P* < 0.001). Conversely, pleasure_*t* − 1_ predicted MIL_*t*_ (*B* = 0.10, *t*[45.35] = 4.68, *P* < 0.001), as did positive affect_*t* − 1_ (*B* = 0.11, *t*[65.64] = 4.16, *P* < 0.001). The above findings suggest a reciprocal relationship between these variables: not only did MIL enhance positive emotions and feelings of pleasure, but also positive emotional experiences fostered a sense of meaning. However, there was no difference in the reactivity of MIL estimates towards affective experiences, including positive affect and pleasure, between groups. Interestingly, when examining time-lagged reactivity across groups, only the MDE/bipolar disorder group had a significantly lower reactivity in terms of how current pleasure_*t* − 1_ predicted MIL_*t*_ (*B* = −0.11, *t*[51.09] = −2.31, *P* = 0.025), and how positive affect_*t* − 1_ predicted MIL_*t*_ (*B* = −0.13, *t*[56.54] = −2.30, *P* = 0.025), as compared with the healthy controls group. This reduced reactivity suggests that, for MDE/bipolar disorder but not for MDD, emotional experiences at previous time points were less potent in regard to predicting a sense of MIL at the next.

## Discussion

To the best of our knowledge, this is the first EMA study to compare the moment-to-moment, cognitive-affective symptom constructs of unipolar versus bipolar depression during MDEs, specifically assessing the bidirectional relationship between positive affect, pleasure/motivation towards current activity and MIL. EMA’s moment-to-moment facility circumvented the recall biases from retrospective self-report measures, allowing us to conduct analyses with time-series data and to integrate the measurements into participants’ daily lives, which enhanced the ecological validity of our study. Referring to our initial hypotheses, while patients with MDD and MDE/bipolar disorder experienced less intense positive affect as compared with healthy controls, the difference was marginally above the significance level. Our momentary data did not suggest significant differences in positive affect dynamics (inertia, variability and instability) across groups. All groups demonstrated a consistent bidirectional relationship between positive affect and current pleasure. Interestingly, we observed that, for patients with MDE/bipolar disorder, increases in current pleasure or positive affect were not as significantly predictive of subsequent MIL as they were for patients with MDD and healthy controls.

The level of positive affect was lower across clinical groups as compared with healthy controls, aligning with previous EMA studies.^14,22,28^ The difference was not statistically significant, which could be due to our limited sample size, and to the fact that our out-patient sample had relatively mild depression severity. Lower positive affect level in clinical groups could underscore deficits in the private experience of positive affect, a core feature of depression. In contrast with our hypotheses, the positive affect dynamics (i.e. inertia, variability and instability) did not significantly differ between patients and healthy controls.To date the existing EMA literature is mixed, with some reporting no significant differences in positive affect dynamics (i.e. variability, instability and inertia) between depressed individuals and healthy controls,^10,14,21,28^ while others reported greater positive affect variability among depressed people as compared with healthy controls.^16,17^ Only one EMA study investigated positive affect dynamics across MDD and bipolar disorder groups during a major depressive episode, which also yielded no significant difference across groups.^22^ This could be explained by several factors. First, positive affect dynamics have been shown to have a non-linear relationship with depressive severity,^29^ suggesting that differences may not emerge uniformly across individuals, regardless of their depression status. For example, previous EMA studies found no significant group difference in positive affect dynamics in people with or without depressive symptoms.^10,14,30^ Second, because all of our clinical group participants were out-patients, the contextual variability introduced by EMA prompts might have contributed to heterogeneity in moment-to-moment findings rather than reflecting differences based solely on clinical diagnoses. Conversely, previous EMA research^18^ found higher positive affect variability in those with bipolar disorder as compared with healthy controls, which we failed to replicate. This could be due to our sample of MDE/bipolar disorder rather than a hypomanic/mixed state. Further EMA research should explore the positive affect dynamics between bipolar disorder and MDD groups during the depressed phase to verify the above findings.

Consistent with previous research,^7^ our findings corroborated a bidirectional, time-lagged relationship between positive affect and pleasure/motivation towards current acitivty (operationalised as anhedonia). This relationship aligned with the NIMH Research Domain Criteria initiative, which identified positive affect as a fundamental component in understanding the symptomatology and neurobiological underpinnings of mental health symptomatology such as anhedonia.^8^ This suggests a potential target for therapeutic intervention, focusing on enhancement of positive affect to mitigate symptoms of anhedonia observed in these disorders.^31^ Such interventions could include tailored cognitive-behavioural strategies and mindfulness practices, which have shown promise in improving affective and cognitive integration in clinical populations.^32^ Our findings support the necessity of personalised therapeutic approaches that consider the unique challenges of emotional regulation in MDE. However, no group differences in the temporal dynamics of positive affect and current pleasure between MDD and MDE/bipolar disorder were found. This aligns with the findings of a recent EMA study^10^ in which individuals with high levels of anhedonia, rather than those in the diagnostic group, differed in regard to positive affect dynamics. This indicates symptom rather than diagnostic group, reflecting the difference in positive affect dynamics, and this observation could be attributed to the methodological approach, which classified anhedonia as a categorical phenomenon that existed along with depression – a present/absent state – rather than as a symptom that existed as a common underlying transdiagnostic psychopathology feature along a continuum. Future research should aim to elucidate the mechanism pertaining to mental health symptomatologies by viewing the underlying dimensions of anhedonia (e.g. its neural and behavioural signatures).^8^

In our study, both clinical groups exhibited a significantly weaker association between MIL and positive affect/current pleasure, as compared with healthy controls. This diminished cognitive control construct could represent the common feature across cognitive-affective symptoms in MDE.^5^ Specifically, for those with MDE/bipolar disorder, increases in positive affect/current pleasure were less effectively predictive of subsequent MIL as compared with healthy controls, albeit those with MDE/bipolar disorder were less likely to be upshifted in cognitive appraisals (MIL) in response to emotional experiences (positive affect/current pleasure) in daily life. This finding highlights the weaker temporal pairing of cognitive and affective symptom constructs in those with MDE/bipolar disorder, which could be elucidated using a biopsychosocial model. Biologically, those with MDE/bipolar disorder tended to exhibit less cognitive-affective synchrony,^5^ diminishing their ability to integrate previous positive emotional experiences into a cohesive sense of self and life purposes.^33^ Psychologically, those with bipolar disorder may have more dysfunctional attitudes^5^ and executive dysfunction,^4^ which thus might hinder the translation of affective well-being (feelings of positive affect/current pleasure) into cognitive well-being (i.e. MIL).^34^ Socially, contextual factors such as interpersonal problems and social rejections impact MIL, with bipolar disorder individuals being particularly susceptible to unstable MIL.^35^ While there is little evidence from our findings that emotional experiences alone characterise the psychopathology of MDE/bipolar disorder, the divergence from response patterns observed in MDD underscores the necessity for distinct therapeutic strategies that address the unique emotional and cognitive integration challenges faced by individuals with MDE/bipolar disorder. Clinically, the predominant focus on pharmacological strategies targeting symptom relief may be insufficient to address emotional dysregulation in MDE/bipolar disorder. Adjunctive psychological treatments, including cognitive behavioural therapy (focusing on the consequences of bipolar disorder for the individual’s sense of self), mindfulness (particularly on awareness and integration of positive experiences) and self-monitoring techniques using EMA – although more extensively studied in MDD – could have substantial clinical significance in improving MDE/bipolar disorder outcomes.^2^ In practical terms, the time-series approach in EMA may offer patient-specific insights into the temporal variations of MDE/bipolar disorder and MDD beyond bimodal measures. This method can enhance the ecological validity of current mood episode assessments and facilitate the development of personalised therapeutic strategies. Our findings instantiate the role of EMA in capturing the pleomorphic positive mental health measures in real time, highlighting its potential to tailor interventions more effectively to individual needs. In the absence of established biomarkers or biosignatures for MDD and MDE/bipolar disorder, EMA enables an ecologically valid profiling that could lead to better decision support in clinical practice in regard to improving quality indicators, self-reported outcomes and the cost-effectiveness of MDD and MDE/bipolar disorder treatment.^36^ Furthermore, the traditional focus in mood disorder research and treatment has predominantly emphasised negative mental health outcomes.^37^ The incorporation of positive mental health measures, such as changes in positive affect and MIL, into diagnostic and therapeutic frameworks could offer more balanced and holistic insights into their well-being.^38^ This approach not only enriches our understanding of the bipolar spectrum but also aligns with a more comprehensive model of mental health that acknowledges the importance of fostering positive outcomes as a pivotal aspect of recovery and resilience in both unipolar and bipolar depression.

Notwithstanding the strength that this is one of the few EMA studies to differentiate bipolar disorder and MDD during MDEs,^22^ and also the first conducted in a Hong Kong Chinese population in this respect, it has several limitations. First, our MDD and MDE/bipolar disorder sample generally comprised out-patients who were relatively clinically stable and with low symptom severity, and therefore may not be representative of those who have more severe symptoms. Second, our modest sample size, due to the stringent inclusion criteria of fulfilling a depressive phase compared with a demographically relatively well-matched control sample, might have precluded us from detecting subtle yet potentially significant group differences in certain EMA measures. However, the total sample size (*N* = 88, consisting of 4632 EMA observations) was relatively large for an EMA study.^10,18,20,22^ Third, while our study captured momentary pleasure/motivation across a variety of real-world contexts, we did not assess whether the tasks performed by participants were activities that they generally liked, disliked or felt neutral about. Future studies should consider including such measures to better understand how activity-dependent factors influence pleasure, motivation and their relationships with other variables. Fourth, while our study focused on state-specific dynamics, we did not examine whether the level of anhedonia (e.g. SHAPS scores) moderated these relationships, which future studies could explore. Fifth, we did not include social interaction, which might provide a more comprehensive understanding of the relationship between affects, anhedonia and the perception of MIL.

Further analysis, by taking into consideration the contexts of social activities and relationships, would provide a better understanding of cognitive-affective symptoms in MDE/bipolar disorder and MDD.^4,5^ Future studies should prioritise the inclusion of lifestyle factors such as sleep, diet and exercise, which are known to play a critical role in emotional regulation and reward processing. For example, disrupted sleep patterns and poor diet have been linked to heightened emotional reactivity and diminished reward sensitivity, both of which are core features of MDEs. Similarly, regular physical activity has been associated with improved mood, increased reward sensitivity and reduced depressive symptoms.^39^ Incorporating these lifestyle factors into research frameworks will provide ecologically valid insights into how they affect reward sensitivity and the core features of MDEs in daily life. By complementing momentary self-reports with multiple digital, psychophysiological measures, as well as evaluating social contexts and cognitive-affective experiences in different well-defined social contexts, a more refined assessment of symptom manifestation in daily life can be conducted, and also the subsequent clarification of the presence of any subtle cognitive-affective symptom temporal pairing deficits between bipolar disorder and MDD during MDEs.

## Data Availability

The data that support the findings of this study are available from the corresponding author upon reasonable request. The authors alone are responsible for the content and writing of the paper.
